# Supplementary material for: Genetic modification of primary human B cells to model high-grade lymphoma
Source: Nat Commun. 2019 Oct 4;10:4543. doi: 10.1038/s41467-019-12494-x (PMC6778131; doi:10.1038/s41467-019-12494-x)
Supplement: Supplementary file 1 — Supplementary Information [file 41467_2019_12494_MOESM1_ESM.pdf]

Genetic modification of primary human B cells to model high-grade lymphoma

Caeser et al.

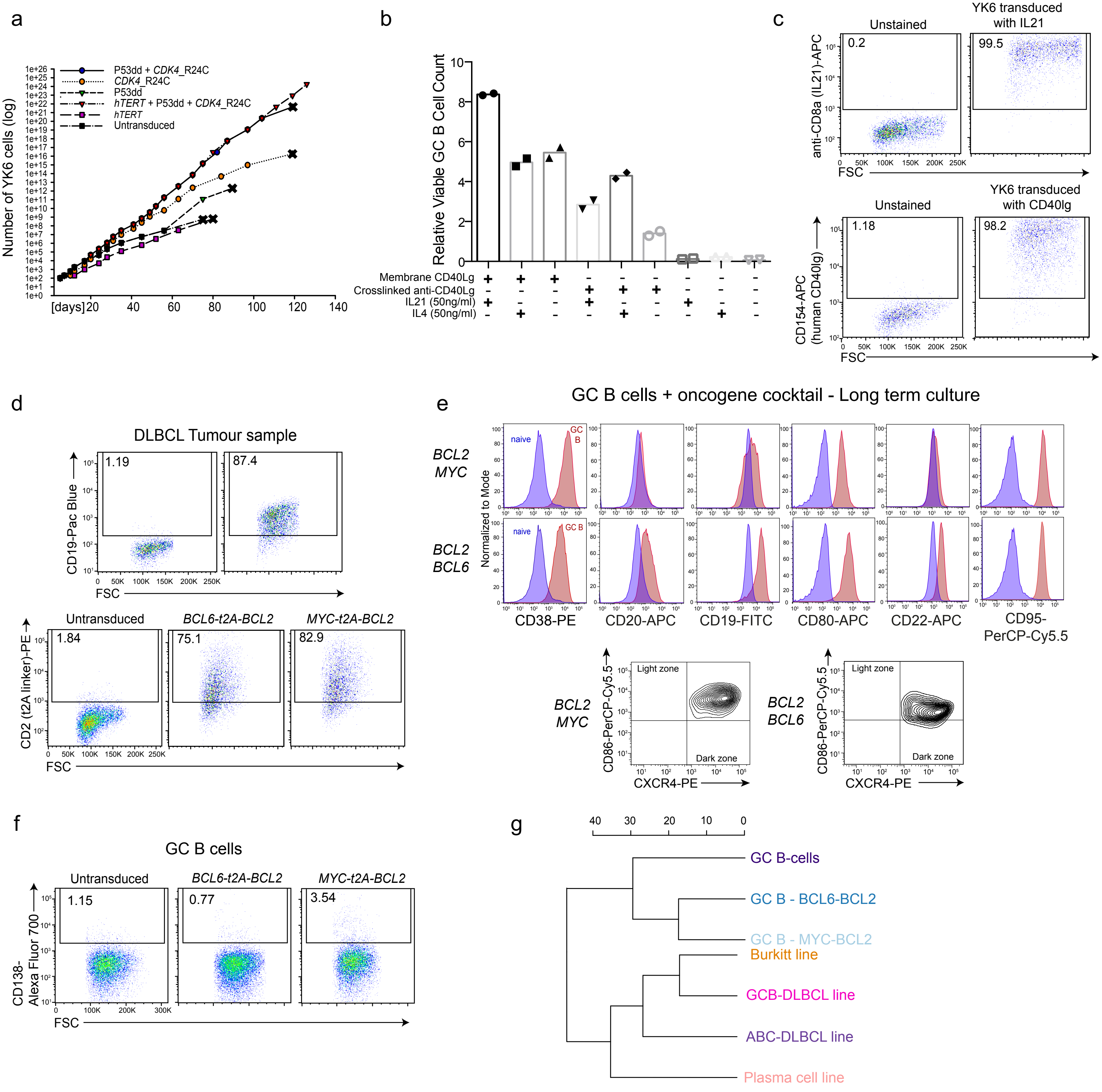

Supplementary Figure 1: In vitro culture of primary human germinal center B cells

(a) Immortalization of the FDC-like feeder cells (YK6) was achieved by retroviral transduction of the indicated oncogenes. Cell number was monitored by manual cell counting. Illustrated is time course showing the number of cells following transduction. Cohorts that had stopped proliferating and underwent replicative senescence are marked with an x at their final timepoint. Source data are provided as a Source Data file.

(b) Primary human B cells were cultured on membrane expressed (HK cells) versus soluble, cross-linked CD40 ligand plus IL21 or IL4. Bar graph shows the relative number of viable cells after 72 hours of stimulation (n=2). Viable cells were determined by flow cytometry and counting beads. Source data are provided as a Source Data file.

(c) Flow cytometry analysis for the expression of CD40lg (CD154) and IL21 (transduction marker CD8a) on immortalized FDC-like cells that were engineered to express CD40lg and IL21. FSC, forward scatter.

(d) DLBCL primary tumor sample was transduced with retroviral vectors expressing *BCL2-t2A-BCL6* and *BCL2-t2A-MYC* using the MuLV-GaLV fusion envelope. Cells were stained for transduction marker CD2 (t2A) and B cell marker CD19 and were analyzed by flow cytometry.

(e) Primary human GC B cells were transduced with the oncogenic cocktail *BCL2-BCL6* and *BCL2-MYC* and cultured long term (Day 73). Representative flow cytometry analysis (n = 3) for the expression of the GC B cell markers CD38, CD20, CD19, CD80, CD22, CD95, CXCR4 and CD86. Red histograms show GC B cells compared to primary human naïve B cells (blue).

(f) Flow cytometry analysis for the expression of CD138 in cultured primary human GC B cells transduced with *BCL2-BCL6* or *BCL2-MYC* was performed 4 weeks after transduction. FSC, forward scatter.

(g) Subtypes of cells in Figure 2e were compared using a signature of germinal center expressed genes (GCB-1). The clustering was based on the Euclidean distance between the average normalized expression.

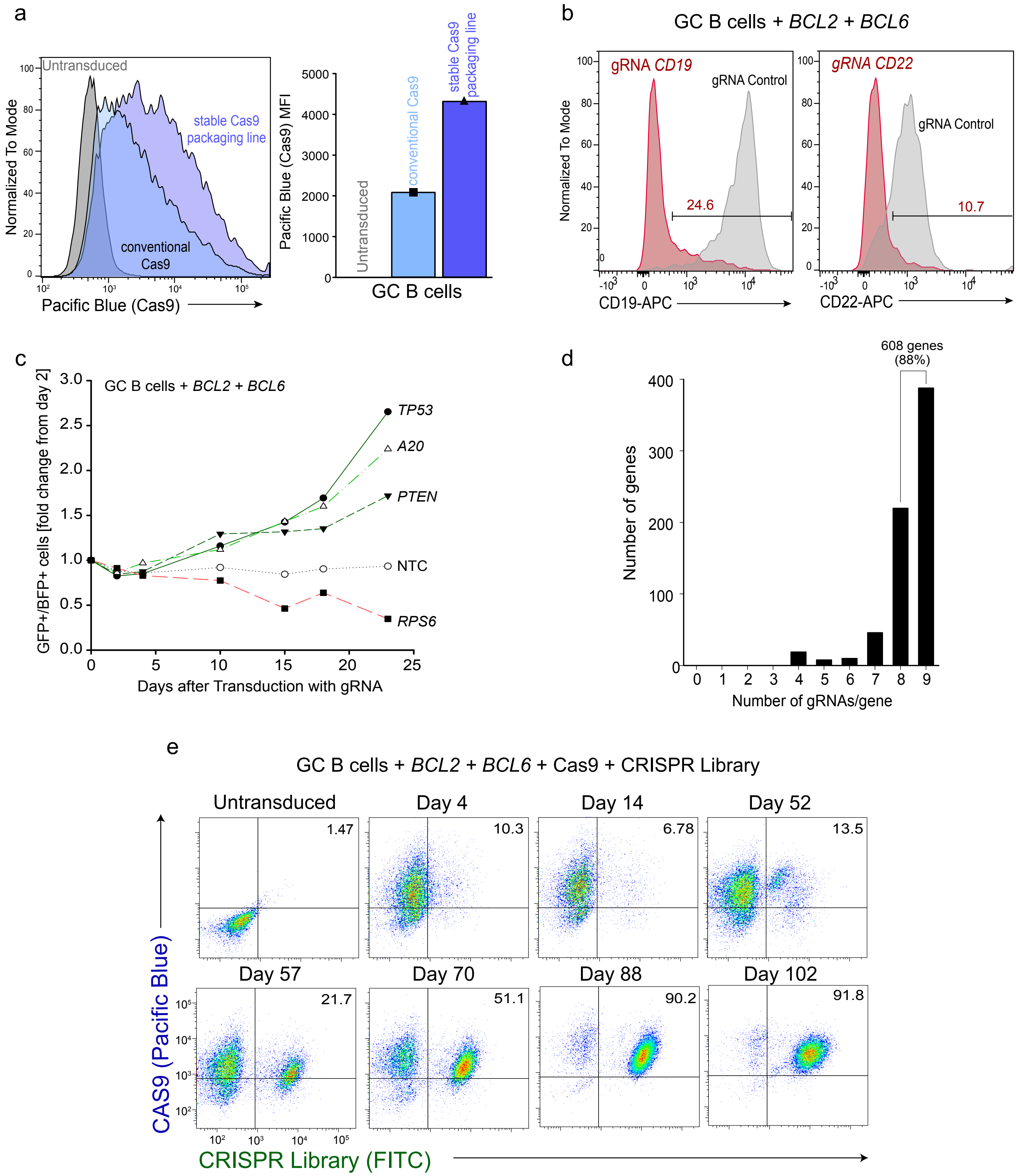

Supplementary Figure 2: CRISPR screening in transduced primary human germinal centre B cells

(a) Two different methods of Cas9 virus production were compared; Conventional transfection of 293T packaging cells with retroviral Cas9 construct was compared to a stable Cas9 packaging line, both tagged with BFP. Bar chart illustrates the mean fluorescence intensity of Pacific Blue (Cas9) in primary GC B cells transduced with each method of Cas9 virus production. Source data are provided as a Source Data file.
(b) Primary GC B cells were transduced with *BCL2-BCL6* and Cas9-BFP and subsequently with gRNAs against *CD19, CD22* and non-targeting control. Staining for CD19 and CD22 was performed 6 days after gRNA transduction and gated on double positive CAS9 (BFP) and gRNA (GFP) expressing cells. Red and grey histograms show CD19/CD22 expression in cells transduced with the indicated gRNA and non-targeting control, respectively.
(c) Primary GC B cells were transduced with *BCL2-BCL6* and Cas9-BFP and subsequently with gRNAs against *TP53, PTEN, A20, RPS6* and non-targeting control (NTC). Following transduction with gRNAs, enrichment or depletion of BFP+GFP+ cells was monitored by flow cytometry. Illustrated is time course showing the relative expansion of BFP+GFP+ cells following transduction. Source data are provided as a Source Data file.
(d) Illumina sequencing revealed the number of gRNAs present per gene in the lymphoma-focused CRISPR library. Source data are provided as a Source Data file.
(e) Flow cytometry analysis showing enrichment of double positive cells transduced by both Cas9-BFP and CRISPR library (GFP) over different timepoints in primary human GC B cells transduced with *BCL2-BCL6*.

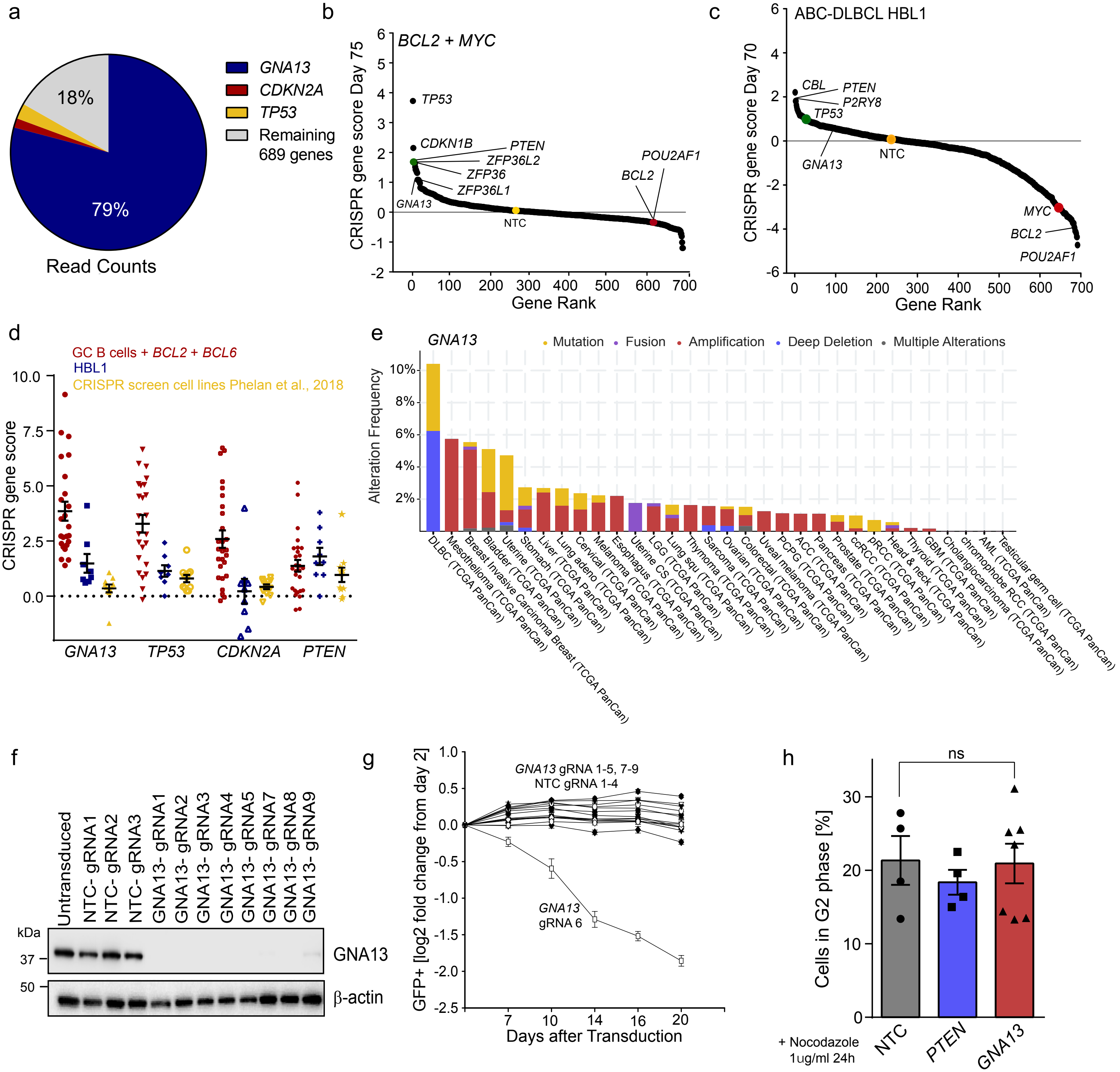

Supplementary Figure 3*: CRISPR screening identifies *GNA13* as a potent tumor suppressor gene*

(a) Normalized read counts for *GNA13* (9 gRNAs), *CDKN2A* (9 gRNAs), *PTEN* (8 gRNAs) compared to counts for the remaining 689 genes in the lymphoma CRISPR library, measured at day 70 of culture.
(b) Rank-ordered depiction of CRISPR gene scores at Day 75 (log2 scale) from highest to lowest (*BCL2-MYC* platform). Selected tumor suppressor genes as well as oncogenes are highlighted in green and red, respectively. Data points above the horizontal line are positively enriched.
(c) HBL1-Cas9 was transduced with the lymphoma-focused CRISPR library. Genes are ranked from highest to lowest according to their CRISPR gene scores at Day 70 (log2 scale). Selected tumor suppressor genes as well as oncogenes are highlighted in green and red, respectively.
(d) CRISPR gene score for *GNA13, TP53, CDKN2A* and *PTEN* in GC B cells transduced with *BCL2* + *BCL6* (n=3, biol), HBL1 (n=1) and compared to published[1] CRISPR gene scores in lymphoma cell lines (n=11). Error bars indicate ± s.e.m of all gRNAs targeting the indicated gene.
(e) TCGA analysis of *GNA13* alteration frequency in different cancer types. Figure obtained from cBioPortal[2,3] (colors were modified for clarity).
(f) The ABC-DLBCL cell line HBL1-Cas9 was transduced with 8 gRNAs against *GNA13* and 4 against non-targeting control. Cells were harvested 10 days after transduction and a western blot performed to validate GNA13 knock-down. β-actin was used as a loading control. Representative of > 3 experiments.
(g) HBL1-Cas9 cells were transduced with 9 *GNA13*, 4 *PTEN* and 4 non-targeting control gRNAs and enrichment or depletion of GFP+ cells was monitored by flow cytometry. Illustrated is time course showing the log2 fold-change relative to baseline (± s.d.) of GFP+ cells following transduction (n = 3). Representative of 3 experiments with 3 replicates/experiment.
(h) Cell Proliferation following *GNA13* and *PTEN* deletion in primary human GC B cells was monitored by Vybrant™ DyeCycle™ Ruby Stain and analyzed by flow cytometry (n=3). GC B cells were treated with Nocodazole (1ug/ml) 24h prior to FACS analysis to arrest cells in G2 phase. Bar chart illustrates G2 positive cells of all gRNAs (*GNA13*=7, *PTEN*=4, NTC=4) for the indicated gene (±s.e.m).

Source data are provided as a Source Data file for a,b,c,d,f,g and h.

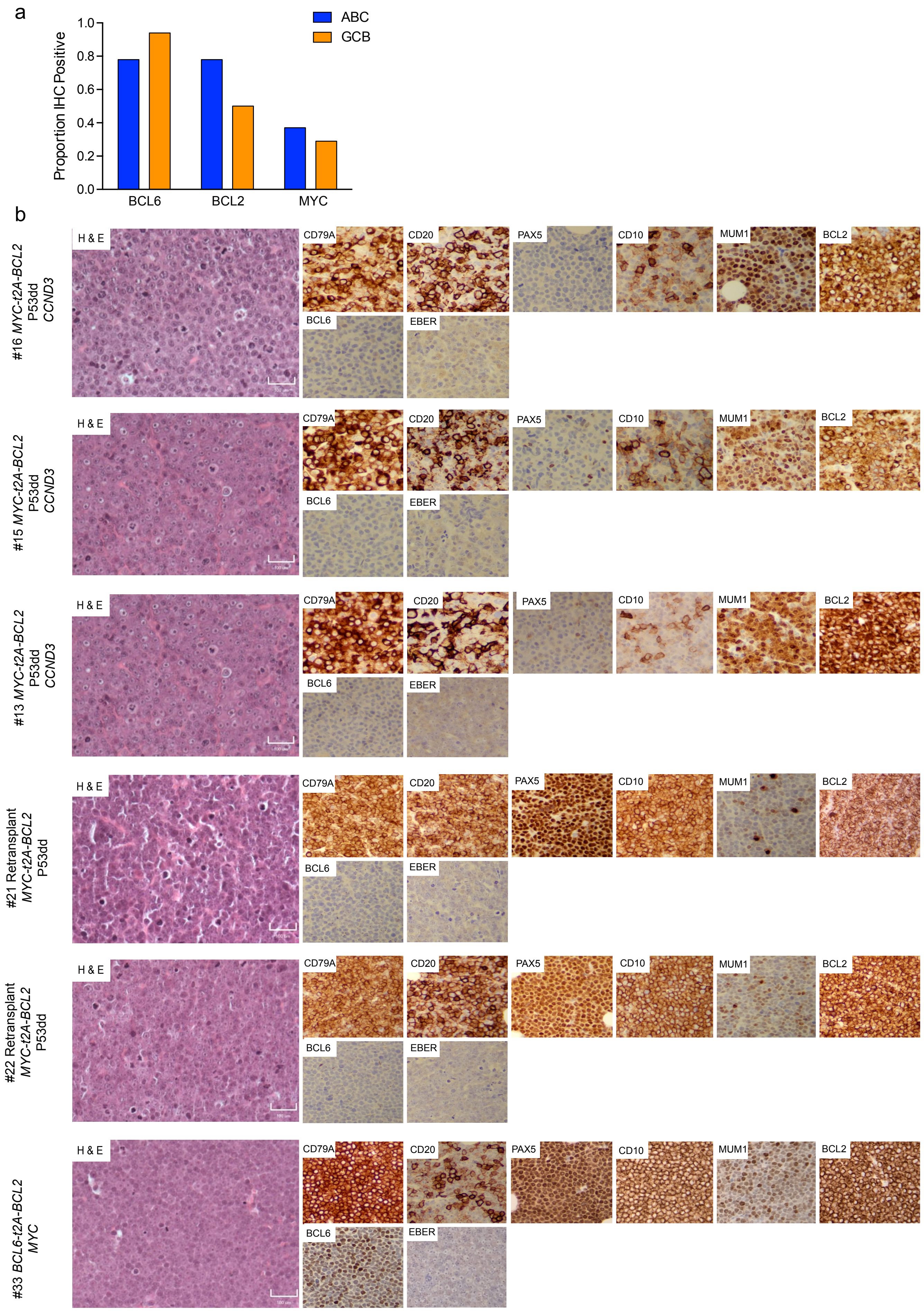

Supplementary Figure 4: Synthetic tumors formed in immunodeficient mice mimic the appearances of human DLBCL

(a) Proportion of human DLBCL biopsies expressing the indicated oncogene (BCL6, BCL2, MYC) assessed by immunohistochemistry in human ABC and GCB-DLBCL samples from patients enrolled in the RICOVER-60 trial; figure adapted from Staiger et al[4]. Source data are provided as a Source Data file. (b) Immunohistochemistry images of synthetic tumors in immunodeficient mice for the indicated markers are shown (Magnification 20x). Six different tumors are shown. Scale bar, 100μM.

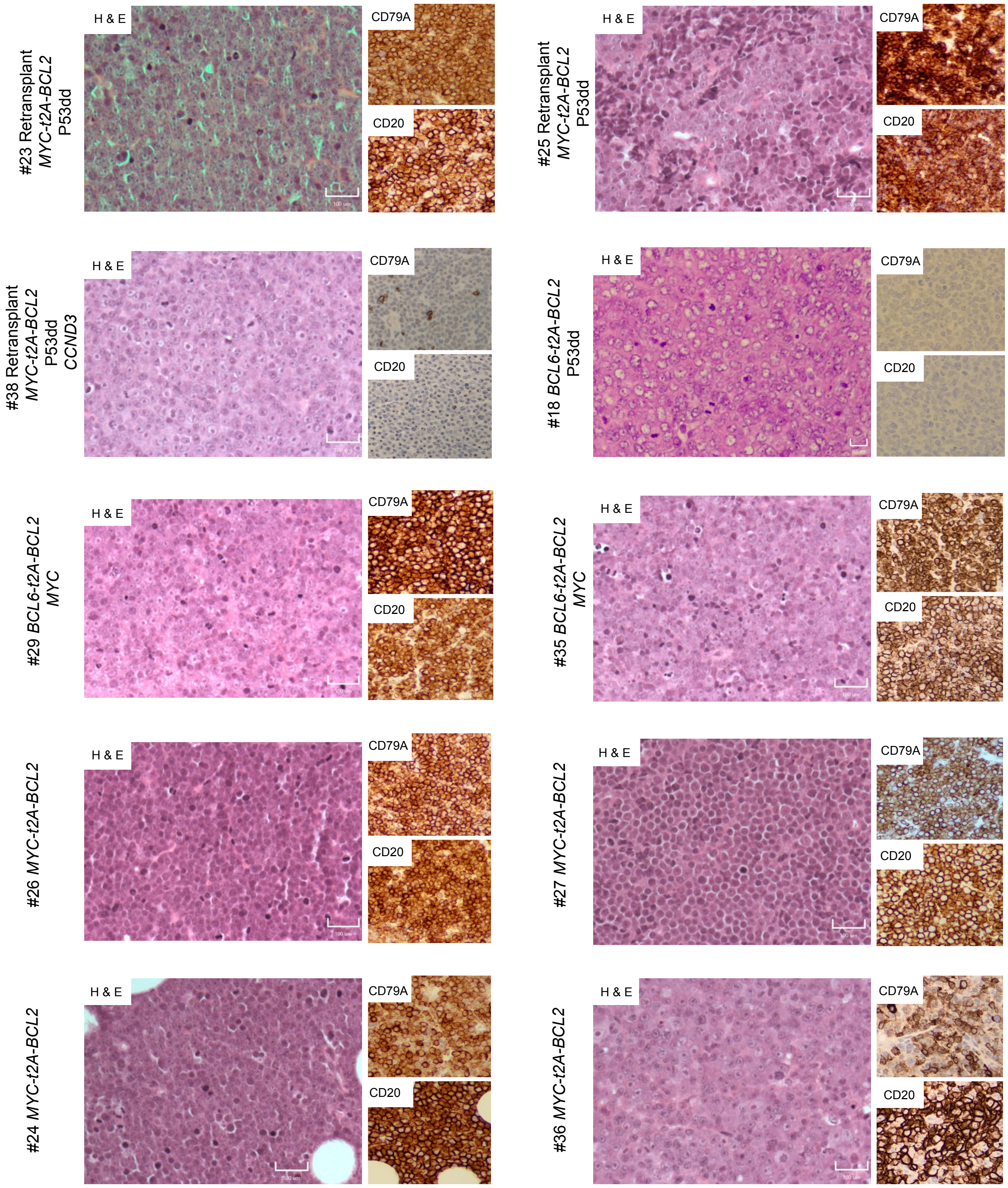

Supplementary Figure 5*:* Synthetic tumors

Immunohistochemistry images for H&E, CD79A and CD20 are shown (Magnification 20x). Ten different tumors are shown. Scale bar, 100μM.

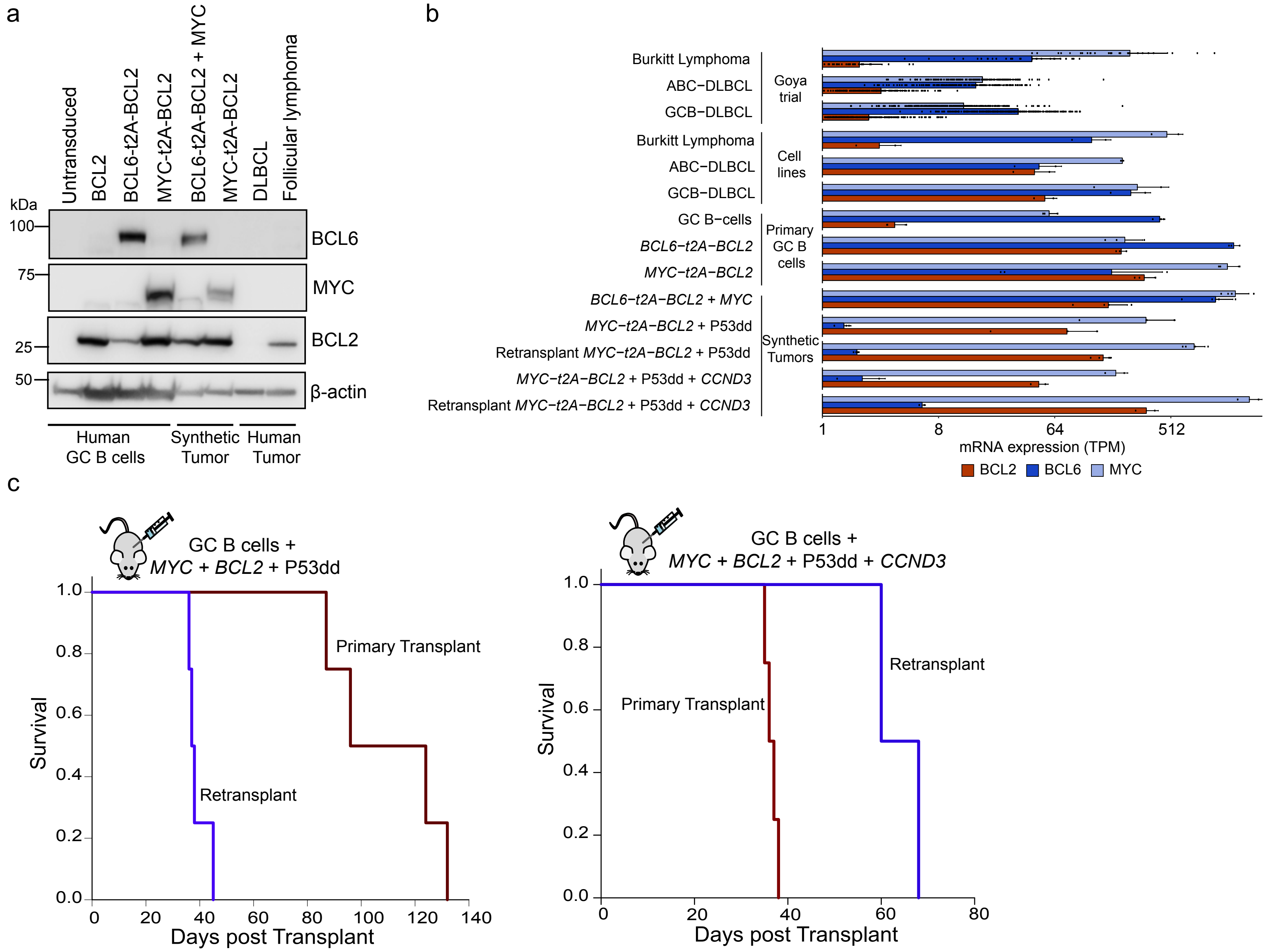

Supplementary Figure 6: BCL6, BCL2 and MYC protein expression in synthetic tumors

(a) Western blot analysis showing BCL6, BCL2 and MYC protein expression in cultured untransduced, transduced (*BCL2, BCL6-t2A-BCL2* or *MYC-t2A-BCL2*) human GC B cells, synthetic tumors (*BCL6-t2A-BCL2+MYC* and *MYC-t2A-BCL2*-transduced human GC B cells) and human tumors (DLBCL and Follicular lymphoma). β-actin was used as a loading control. Source data are provided as a Source Data file.
(b) Bar chart showing mean mRNA expression levels (TPM, ± s.d.) for BCL2 (red), BCL6 (dark blue) and MYC (light blue) in human patient samples (GOYA trial, n=504), lymphoma cell lines (Burkitt lymphoma n=2 (Raji, Mutu), ABC-DLBCL n=2 (TMD8, HBL1), GCB-DLBCL n=2 (SUDHL4, DOHH2)), primary GC B cells (untransduced n=3, *BCL6-t2A-BCL2* n=3, *MYC-t2A-BCL2* n=3) and synthetic tumors (*BCL6-t2A-BCL2+MYC* n=4, *MYC-t2A-BCL2*+P53dd n=3, Retransplant *MYC-t2A-BCL2*+P53dd n=4, *MYC-t2A-BCL2*+P53dd+*CCND3* n=3, Retransplant *MYC-t2A-BCL2*+P53dd+*CCND3* n=2).
(c) Primary human GC B cells harvested from primary tumors were retransplanted subcutaneously into NOD/SCID/gamma mice and monitored for palpable tumors. Mice were culled when tumors reached 12mm in size. Overall survival of the recipient mice (Primary transplant and Retransplant) is plotted as a Kaplan-Meier curve. Source data are provided as a Source Data file.

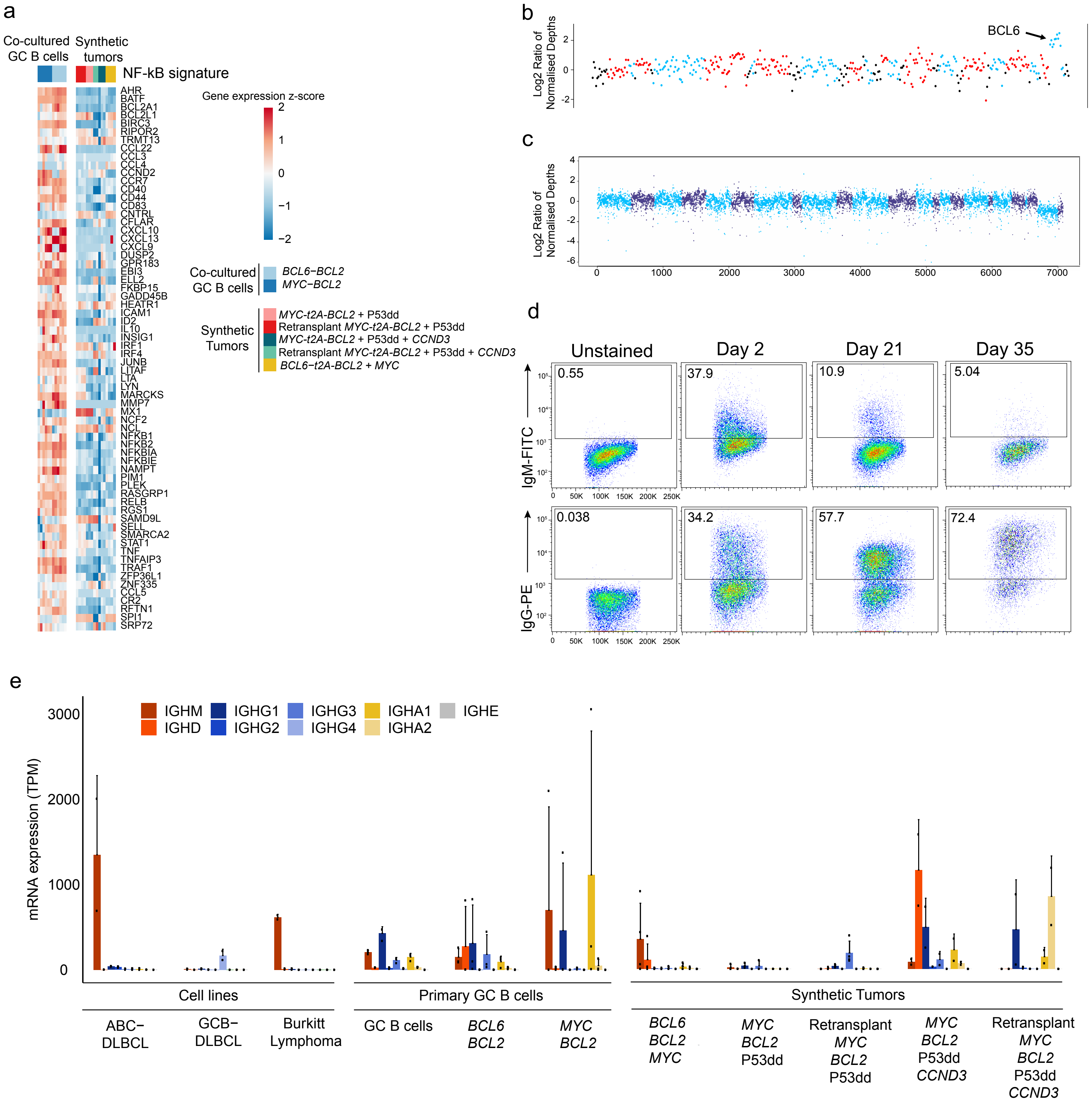

Supplementary Figure 7: Phenotypic and molecular characterization of synthetic tumors

(a) Heat map of gene expression of NF-kB signaling signatures (Supplementary Table 1) in co-cultured Germinal Centre B-cells (n=12) and synthetic tumors (*BCL6-t2A-BCL2+MYC* n=4, *MYC-t2A-BCL2*+P53dd n=3, Retransplant *MYC-t2A-BCL2*+P53dd n=4, *MYC-t2A-BCL2*+P53dd+*CCND3* n=3, Retransplant *MYC-t2A-BCL2*+P53dd+*CCND3* n=2).
(b) Gains corresponding to BCL6 on chr3 are shown. Black points are background regions whilst red and blue features correspond to genes analysed for their copy number state. BCL6 is indicated by arrow.
(c) Copy number across whole genome showing no evidence of aneuploidy. Alternating colors correspond to different chromosomes. The x-axis represents bin index rather than absolute genomic coordinate.
(d) Flow cytometry analysis for the expression of IgM and IgG in human GC B cells transduced with *BCL6*-t2A-*BCL2* over three different timepoints. FSC, forward scatter.
(e) Bar chart showing mRNA expression levels (TPM, ± s.d.) for immunoglobulin heavy chain constant regions, IgM (red), IgD (orange), IgG1-4 (different shades of blue), IgA1-2 (different shades of yellow) and IgE (grey) in lymphoma cell lines (Burkitt lymphoma n=2 (Raji, Mutu), ABC-DLBCL n=2 (TMD8, HBL1), GCB-DLBCL n=2 (SUDHL4, DOHH2)), primary GC B cells (untransduced n=3, *BCL6-t2A-BCL2* n=3, *MYC-t2A-BCL2* n=3) and synthetic tumors (*BCL6-t2A-BCL2+MYC* n=4, *MYC-t2A-BCL2*+P53dd n=3, Retransplant *MYC-t2A-BCL2*+P53dd n=4, *MYC-t2A-BCL2*+P53dd+*CCND3* n=3, Retransplant *MYC-t2A-BCL2*+P53dd+*CCND3* n=2).

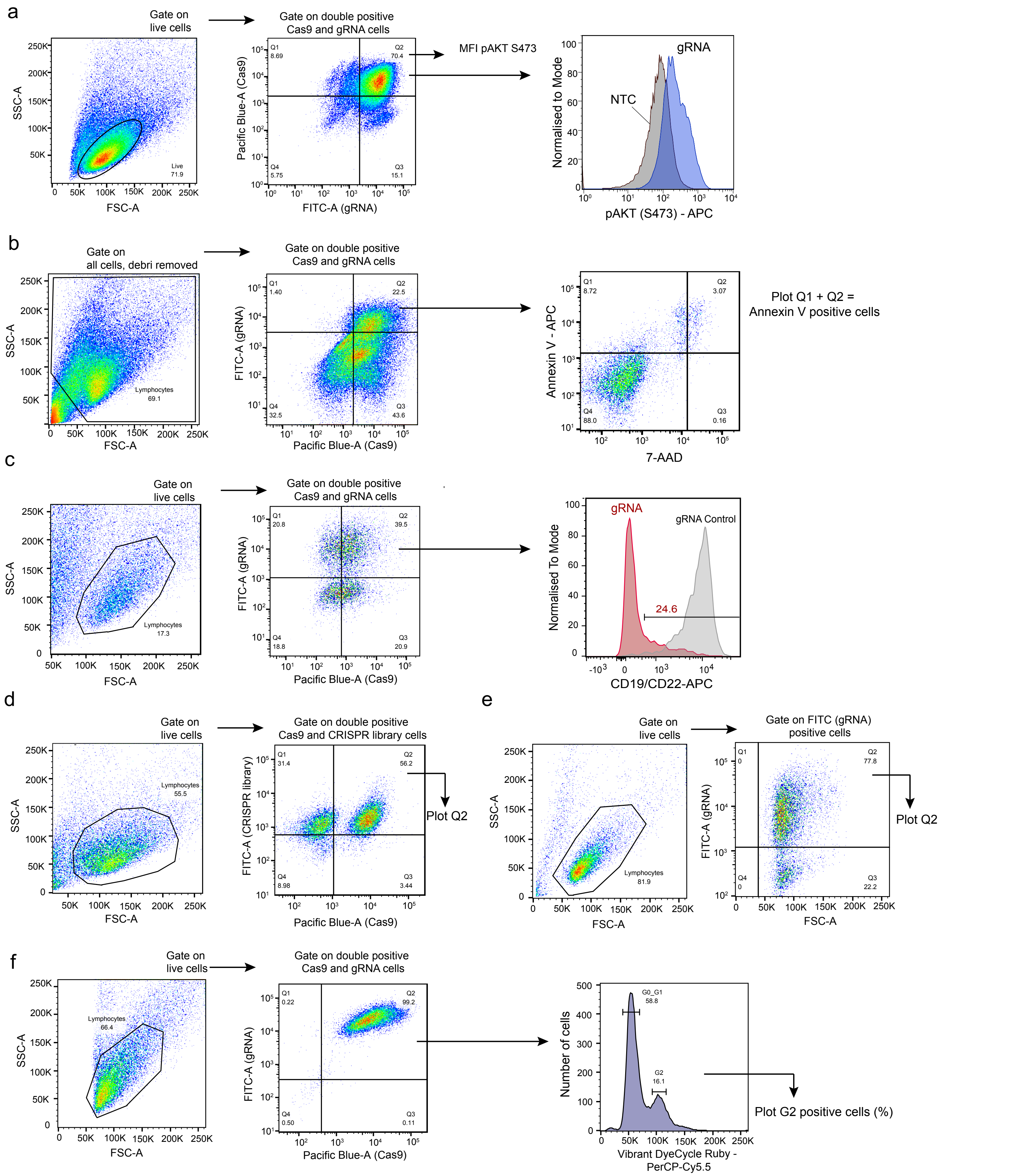

Supplementary Figure 8*:* Gating Strategy

FACS gating strategy for
(a) live, BFP+GFP+ (Cas9/gRNA) expressing pAKT (S473) cells as seen in Figure 4b.
(b) live, BFP+GFP+ (Cas9/gRNA) expressing cells also positive for Annexin V as seen in Figure 4c.
(c) live, BFP+GFP+ (Cas9/gRNA) expressing cells with knockdown of CD19, CD22 or control as seen in Supplementary Figure 2b.
(d) live, BFP+GFP+ (Cas9/CRISPR library) expressing cells as seen in Supplementary Figure 2c.
(e) live, GFP+ (gRNA) expressing cells as seen in Supplementary Figure 3g.
(f) live, BFP+GFP+ (Cas9/gRNA) expressing cells with G2 cell cycle analysis as seen in Supplementary Figure 3h.

Signature name	Figure	Description	Reference
CC-2	Figure 2 e	Genes 1.33x more highly expressed in human and mouse centrocytes than centroblasts	https://lymphochip.nih.gov/signaturedb/
GCB-1	Figure 2 e	Fig. 3b Dave et al. NEJM 354:2431 (2006)	https://lymphochip.nih.gov/signaturedb/
BCRUp-2	Figure 2 e	Signaling pathway	https://lymphochip.nih.gov/signaturedb/
MYCUp-2	Figure 2 e	Myc_overexpression_1.5x_up	https://lymphochip.nih.gov/signaturedb/
FL1	Figure 2 e	Fig. 3 Dave et al. NEJM 351:2159 (2004)	https://lymphochip.nih.gov/signaturedb/
STAT3Up-1	Figure 2 e	Fig. 2e Lam et al. Blood. 2008 111(7):3701-13.	https://lymphochip.nih.gov/signaturedb/
GCB	Supplementary Figure 6 b	Gene expression classifier that distinguish GCB-DLBCL and ABC-DLBCL; Genes enriched in GCB-DLBCL	Dave et al. Cell 2017
ABC	Supplementary Figure 6 b	Gene expression classifier that distinguish GCB-DLBCL and ABC-DLBCL; Genes enriched in ABC-DLBCL	Dave et al. Cell 2017
MHG Up	Supplementary Figure 6 b	Genes upregulated in Molecular High Grade DLBCL	Sha et al. Genome Medicine 2015
MHG Down	Supplementary Figure 6 b	Genes downregulated in Molecular High Grade DLBCL	Sha et al. Genome Medicine 2015
DHIT Up	Supplementary Figure 6 b	Genes upregulated in Double Hit DLBCL; Top 10 with the highest DHITsig Importance Score	Ennishi et al. Journal of Clinical Oncology 2019
DHIT Down	Supplementary Figure 6 b	Genes downregulated in Double Hit DLBCL; Top 10 with the lowest DHITsig Importance Score	Ennishi et al. Journal of Clinical Oncology 2019
NFKB signature	Supplementary Figure 7 a	Union of genes from NFkB-1, NFkB-2, NFkB-3, NFkB-4, NFkB-5 signatures	https://lymphochip.nih.gov/signaturedb/

Supplementary Table 1 Summary of gene signatures used in gene expression profiles.

Oncogenic Cocktail	Tumor	V gene	J gene	CDR3	V gene mutations	J gene mutations	V-REGION identity nt	J-REGION identity nt
MYC / BCL2 / TP53dd / CCND3	Primary	IGHV3-30*03 F, IGHV3-30*18 F, IGHV3-30-5*01 F	IGHJ6*02 F	CAKEDYYGLGALDVW	90.09	69.57	200/222 nt	32/46 nt
MYC / BCL2 / TP53dd / CCND3	Primary	IGHV3-30-3*01 F	IGHJ4*02 F	CARDQSRGYPFDYW	87.84	90	195/222 nt	27/30 nt
MYC / BCL2 / TP53dd / CCND3	Retransplant	IGHV3-30-3*01 F	IGHJ4*02 F	CARDQSRGYPFDYW	87.78	93.55	194/221 nt	29/31 nt
MYC / BCL2 / TP53dd / CCND3	Retransplant	IGHV3-30-3*01 F	IGHJ4*02 F	CARDQSRGYPFDYW	87.78	93.55	194/221 nt	29/31 nt
MYC / BCL2 / TP53dd	Primary	IGHV3-30*03 F, IGHV3-30*18 F, IGHV3-30*19 F, IGHV3-30-5*01 F, IGHV3-33*05 F	IGHJ4*02 F	CAKGYDYVWGSYRYDFDYW	84.93	93.55	186/219 nt	29/31 nt
MYC / BCL2 / TP53dd	Primary	IGHV3-30*03 F, IGHV3-30*18 F, IGHV3-30-5*01 F	IGHJ4*02 F	CAKGYDYVWGSYRYDFDYW	97.74	93.55	216/221 nt	29/31 nt
MYC / BCL2 / TP53dd	Retransplant	IGHV3-30*03 F, IGHV3-30*18 F, IGHV3-30*19 F, IGHV3-30-5*01 F, IGHV3-33*05 F	IGHJ4*02 F	CAKGYDYVWGSYRYDFDYW	84.93	93.55	186/219 nt	29/31 nt
MYC / BCL2 / TP53dd	Retransplant	IGHV3-30*03 F, IGHV3-30*18 F, IGHV3-30*19 F, IGHV3-30-5*01 F, IGHV3-33*05 F	IGHJ4*02 F	CAKGYDYVWGSYRYDFDYW	84.93	93.55	186/219 nt	29/31 nt
MYC / BCL2 / BCL6	Primary	IGHV3-30*01 F, IGHV3-30-3*01 F	IGHJ4*02 F	CARVSSAYDLPKYYFDYW	91.4	93.55	202/221 nt	29/31 nt
MYC / BCL2 / BCL6	Primary	IGHV1-2*06 F	IGHJ6*03 F	CARDIVVVPAAGPSYYYYMDVW	100	89.13	219/219 nt	41/46 nt
MYC / BCL2 / BCL6	Primary	IGHV4-34*12 F	IGHJ6*03 F	CARGSLKYNYNMDVW	93.12	80.43	203/218 nt	37/46 nt
MYC / BCL2 / BCL6	Primary	IGHV3-43D*04 F	IGHJ4*02 F	VHPIVVVPAAMGPGE#YFDYW	94.57	90.32	209/221 nt	28/31 nt
Healthy Donor 1	No clusters larger than 1.0% repertoire							
Healthy Donor 2								

Supplementary Table 2 Table for V and J segment mutations and identity (nt) shown for primary tumor samples (n=8) and retransplants (n=4).

Supplementary References

1 Phelan, J. D. *et al.* A multiprotein supercomplex controlling oncogenic signalling in lymphoma. *Nature* **560**, 387-391, doi:10.1038/s41586-018-0290-0 (2018).

2 Gao, J. *et al.* Integrative analysis of complex cancer genomics and clinical profiles using the cBioPortal. *Science signaling* **6**, pl1, doi:10.1126/scisignal.2004088 (2013).

3 Cerami, E. *et al.* The cBio cancer genomics portal: an open platform for exploring multidimensional cancer genomics data. *Cancer Discov* **2**, 401-404, doi:10.1158/2159-8290.Cd-12-0095 (2012).

4 Staiger, A. M. *et al.* Clinical Impact of the Cell-of-Origin Classification and the MYC/ BCL2 Dual Expresser Status in Diffuse Large B-Cell Lymphoma Treated Within Prospective Clinical Trials of the German High-Grade Non-Hodgkin's Lymphoma Study Group. *Journal of clinical oncology : official journal of the American Society of Clinical Oncology* **35**, 2515-2526, doi:10.1200/JCO.2016.70.3660 (2017).
