# Supplementary material for: Genetic modification of primary human B cells to model high-grade lymphoma
Source: Nat Commun. 2019 Oct 4;10:4543. doi: 10.1038/s41467-019-12494-x (PMC6778131; doi:10.1038/s41467-019-12494-x)
Supplement: Supplementary file 3 — Description of Additional Supplementary Files [file 41467_2019_12494_MOESM3_ESM.pdf]

Description of Additional Supplementary Files

File Name: Supplementary Movie 1

Description: Movie shows culture of GC B cells alone from the time of plating to 132 hours after. Scale bar, 50µM.

File Name: Supplementary Movie 2

Description: Movie shows culture of YK6 cells alone from the time of plating to 132 hours after. Scale bar, 50µM.

File Name: Supplementary Movie 3

Description: Movie shows culture of YK6 + GC B cells from the time of plating to 132 hours after. Scale bar, 50µM.

File Name: Supplementary Movie 4

Description: Movie shows culture of YK6-CD40Lg + GC B cells from the time of plating to 132 hours after. Scale bar, 50µM.

File Name: **Supplementary Movie 5**

Description: Movie shows culture of YK6-CD40Lg-IL21 + GC B cells from the time of plating to 132 hours after. Scale bar, 50µM.

File Name: Supplementary Data 1

Description: Enrichment scores based on relative read counts of barcoded expression constructs for transcription factors or their mutant versions in GC B cells co-transduced with *BCL2* over 4 different timepoints (n=3).

File Name: Supplementary Data 2

Description: CRISPR gene scores shown for GC B cells transduced with *BCL2* & *BCL6* (n=3), *BCL2* & *MYC* (n=1) and cell line HBL1 (n=1) with the CRISPR library targeting 692 genes.

File Name: Supplementary Data 3

Description: Protein altering variants identified by MUTEC2 using the matched, pre-transduced, germinal center B cells as the normal control.
