# Supplementary material for: Genetic modification of primary human B cells to model high-grade lymphoma
Source: Nat Commun. 2019 Oct 4;10:4543. doi: 10.1038/s41467-019-12494-x (PMC6778131; doi:10.1038/s41467-019-12494-x)
Supplement: Supplementary file 12 — Reporting Summary [file 41467_2019_12494_MOESM12_ESM.pdf]

nature research

Corresponding author(s): Daniel Hodson

Last updated by author(s): Sep 6, 2019

Reporting Summary

Nature Research wishes to improve the reproducibility of the work that we publish. This form provides structure for consistency and transparency in reporting. For further information on Nature Research policies, see Authors & Referees and the Editorial Policy Checklist.

Statistics

For all statistical analyses, confirm that the following items are present in the figure legend, table legend, main text, or Methods section.

n/a	Confirmed	
☐	☒	The exact sample size (*n*) for each experimental group/condition, given as a discrete number and unit of measurement
☐	☒	A statement on whether measurements were taken from distinct samples or whether the same sample was measured repeatedly
☐	☒	The statistical test(s) used AND whether they are one- or two-sided<br>*Only common tests should be described solely by name; describe more complex techniques in the Methods section.*
☒	☐	A description of all covariates tested
☒	☐	A description of any assumptions or corrections, such as tests of normality and adjustment for multiple comparisons
☐	☒	A full description of the statistical parameters including central tendency (e.g. means) or other basic estimates (e.g. regression coefficient) AND variation (e.g. standard deviation) or associated estimates of uncertainty (e.g. confidence intervals)
☐	☒	For null hypothesis testing, the test statistic (e.g. F, t, r) with confidence intervals, effect sizes, degrees of freedom and P value noted<br>*Give P values as exact values whenever suitable.*
☒	☐	For Bayesian analysis, information on the choice of priors and Markov chain Monte Carlo settings
☒	☐	For hierarchical and complex designs, identification of the appropriate level for tests and full reporting of outcomes
☒	☐	Estimates of effect sizes (e.g. Cohen's *d*, Pearson's *r*), indicating how they were calculated

Our web collection on statistics for biologists contains articles on many of the points above.

Software and code

Policy information about availability of computer code

Data collection	BD LSR2, BD Fortessa, BD ARIA ,Azure Biosystem (c300), Olympus Microscope
Data analysis	Flowjo 10.0.8.0, SigmaPlot 12.5, Graphpad Prism 6

For manuscripts utilizing custom algorithms or software that are central to the research but not yet described in published literature, software must be made available to editors/reviewers. We strongly encourage code deposition in a community repository (e.g. GitHub). See the Nature Research guidelines for submitting code & software for further information.

Data

Policy information about availability of data

All manuscripts must include a data availability statement. This statement should provide the following information, where applicable:

- Accession codes, unique identifiers, or web links for publicly available datasets
- A list of figures that have associated raw data
- A description of any restrictions on data availability

The authors declare that the data supporting the findings of this study are available within the paper and its supplementary information.

Gene expression data has been uploaded to the EGA database under the accession number EGAS00001003560 [https://www.ebi.ac.uk/ega/datasets/EGAD00001005281]. Deep sequencing of PCR amplified immunoglobulin heavy chain variable gene regions has been submitted to the SRA under the BioProject ID: PRJNA551148 [https://www.ncbi.nlm.nih.gov/Traces/study/?acc=PRJNA551148]. All the other data supporting the findings of this study are available within the article and its supplementary information files and from the corresponding author upon reasonable request. A reporting summary for this article is available as a Supplementary Information file.

Field-specific reporting

Please select the one below that is the best fit for your research. If you are not sure, read the appropriate sections before making your selection.

☒ Life sciences ☐ Behavioural & social sciences ☐ Ecological, evolutionary & environmental sciences

For a reference copy of the document with all sections, see nature.com/documents/nr-reporting-summary-flat.pdf

Life sciences study design

All studies must disclose on these points even when the disclosure is negative.

Sample size	No samples-size calculation was performed. For in vitro studies, a minimum of n=3 (independent biological replicates) was used if not otherwise stated in the figure legend.<br>For in vivo mouse experiments, we used n=3-4 per treatment cohort.
Data exclusions	No data was excluded from analysis.
Replication	All attempts at replication were successful.
Randomization	For in vitro experiments, donor-derived cells were paired and distributed into control and experimental group for comparison.<br>For in vivo experiments, age- and sex-matched mice were randomly allocated for cohorts.
Blinding	The investigators were not blinded as experimental conditions required us to know the identity of samples and donors. Animal husbandry technicians providing guidance on when to sacrifice animals were blinded.

Reporting for specific materials, systems and methods

We require information from authors about some types of materials, experimental systems and methods used in many studies. Here, indicate whether each material, system or method listed is relevant to your study. If you are not sure if a list item applies to your research, read the appropriate section before selecting a response.

Materials & experimental systems

n/a	Involved in the study	
☐	☒	Antibodies
☐	☒	Eukaryotic cell lines
☒	☐	Palaeontology
☐	☒	Animals and other organisms
☒	☐	Human research participants
☐	☒	Clinical data

Methods

n/a	Involved in the study	
☒	☐	ChIP-seq
☐	☒	Flow cytometry
☒	☐	MRI-based neuroimaging

Antibodies

Antibodies used	All antibodies used in this study are commercially available and have been validated by the manufacturer.<br>A complete list of antibodies, including catalog number, is provided in Methods.
Validation	The following Western Blot antibodies were used:<br>#4970 Beta-actin (13E5, Cell Signalling Technology), 1:10000, Supplementary Figure 3f & 6a<br>ab128900 GNA13 (EPR5436, Abcam), 1:1000, Supplementary Figure 3f<br>sc-764 c-myc (N-262, Santa Cruz), 1:500, Supplementary Figure 6a<br>BCL-6 (D65C10, Cell Signaling Technology #5650), 1:500, Supplementary Figure 6a<br>BCL-2 (610539, Becton Dickinson Biosciences), 1:500, Supplementary Figure 6a<br><br>The following FACS antibodies were used:<br>Anti-human CD38 (HB7), #12-0388-42, 1:500<br>Anti-human CD20 (2H7), #17-0209-41, 1:500<br>Anti-human CD19 (HIB19), #302223/302211/302205, 1:500<br>Anti-human CD10 (97C5), MiltenyiBiotec, 130-093-450, 1:500<br>Anti-human CD2 (RPA-2.10), #300207/300235, 1:500<br>Anti-mouse CD90.1 Thy1.1 (OX-7), #202529, 1:500<br>Anti-human CD154 (24-31), #310805, 1:500<br>Anti-mouse CD8a (53-6.7), #100712, 1:500<br>Anti-human CD22 (HIB22), #302510, 1:500<br>Anti-human CD80 (2D10), #305219, 1:500

	Anti-human CD95 (DX2), #305629, 1:500<br>Anti-human CD86 (IT2.2), #305419, 1:500<br>Anti-human CXCR4 (12G5), #306505, 1:500<br>Anti-human IgM (MHM-88), #314506, 1:500<br>Anti-human IgG #H10164, 1:500<br>#11962 Phospho-Akt (Ser473) (D9E) XP Rabbit mAb (Cell Signalling)<br><br>All antibodies were purchased from Biolegend if not otherwise stated.

Eukaryotic cell lines

Policy information about cell lines

Cell line source(s)	Obtained from Dr Louis Staudt: HBL1, BJAB, U2932, TMD8, SUDHL4, DOHH2, Raji, Mutu.<br>NCIH929 obtained from ATCC CRL-9068.<br>Lenti-X 293T (Clontech Laboratories, 632180)
Authentication	Identity was verified using a 16-amplicon multiplexed copy number variant fingerprinting assay.
Mycoplasma contamination	All cell lines used in this study were confirmed to be free from mycoplasma contamination.
Commonly misidentified lines<br>(See ICLAC register)	No commonly misidentified cell lines were used in these studies.

Animals and other organisms

Policy information about studies involving animals; ARRIVE guidelines recommended for reporting animal research

Laboratory animals	All animals used were NSG (non-obese diabetic/severe combined immunodeficient/common gamma chain deficient). Specific numbers and groups of animals are provided in figure legends accordingly.
Wild animals	No wild animals were utilized during the course of these studies.
Field-collected samples	No field-collected samples were utilized during the course of these studies.
Ethics oversight	This research has been regulated under the Animals (Scientific Procedures) Act 1986 Amendment Regulations 2012 following ethical review by the University of Cambridge Animal Welfare and Ethical Review Body (AWERB).

Note that full information on the approval of the study protocol must also be provided in the manuscript.

Clinical data

Policy information about clinical studies

All manuscripts should comply with the ICMJE guidelines for publication of clinical research and a completed CONSORT checklist must be included with all submissions.

Clinical trial registration	*Provide the trial registration number from ClinicalTrials.gov or an equivalent agency.*
Study protocol	*Note where the full trial protocol can be accessed OR if not available, explain why.*
Data collection	*Describe the settings and locales of data collection, noting the time periods of recruitment and data collection.*
Outcomes	*Describe how you pre-defined primary and secondary outcome measures and how you assessed these measures.*

Flow Cytometry

Plots

Confirm that:

☒ The axis labels state the marker and fluorochrome used (e.g. CD4-FITC).

☒ The axis scales are clearly visible. Include numbers along axes only for bottom left plot of group (a 'group' is an analysis of identical markers).

☒ All plots are contour plots with outliers or pseudocolor plots.

☒ A numerical value for number of cells or percentage (with statistics) is provided.

Methodology

Sample preparation	Cells were prepared as follows unless otherwise stated in Methods:<br>Cells used in co-cultures assays were gently de-attached from feeders by pipetting, with care used to not remove feeders,

collected and washed (500 x g, 5 min) several times with PBS and filtered before staining for relevant markers. For analysis, cells were resuspended in PBS with 2% FBS.

Instrument	BD Biosciences LSR2, BD Fortessa, BD Biosciences Aria2
Software	BD FACS Diva 6.1.3 software was used to collect flow cytometry data. FlowJo 10.0.8 software was used for the analysis of flow cytometry data.
Cell population abundance	Purity of magnetic-bead purified GC B cells were analyzed by staining for CD38, CD20, CD10 and CD19. Generally, more than 10,000 events were collected.
Gating strategy	Live cells were gated using FSC/SSC parameters. Positive cells for a marker were gated using nontransduced negative controls. Gating strategy can be found in Supplementary Figure 8.

☒ Tick this box to confirm that a figure exemplifying the gating strategy is provided in the Supplementary Information.
